# Exploring the deep learning of artificial intelligence in nursing: a concept analysis with Walker and Avant’s approach

**DOI:** 10.1186/s12912-024-02170-x

**Published:** 2024-08-01

**Authors:** Supichaya Wangpitipanit, Jiraporn Lininger, Nick Anderson

**Affiliations:** 1 Visiting Assistant Professor, Division of Health Informatics, Department of Public Health Sciences, UC Davis School of Medicine, University of California, Davis, USA, Division of Community Health Nursing, Ramathibodi School of Nursing, Faculty of Medicine Ramathibodi Hospital, Mahidol University, Bangkok, Thailand; 2 Division of Community Health Nursing, Ramathibodi School of Nursing, Faculty of Medicine Ramathibodi Hospital, Mahidol University, Bangkok, Thailand; 3 Division of Health Informatics, Department of Public Health Sciences, UC Davis School of Medicine, University of California, Davis, USA

**Keywords:** Deep learning, Artificial intelligence, Nursing, Concept analysis, Walker and Avant

## Abstract

**Background:**

In recent years, increased attention has been given to using deep learning (DL) of artificial intelligence (AI) in healthcare to address nursing challenges. The adoption of new technologies in nursing needs to be improved, and AI in nursing is still in its early stages. However, the current literature needs more clarity, which affects clinical practice, research, and theory development. This study aimed to clarify the meaning of deep learning and identify the defining attributes of artificial intelligence within nursing.

**Methods:**

We conducted a concept analysis of the deep learning of AI in nursing care using Walker and Avant’s 8-step approach. Our search strategy employed Boolean techniques and MeSH terms across databases, including BMC, CINAHL, ClinicalKey for Nursing, Embase, Ovid, Scopus, SpringerLink and Spinger Nature, ProQuest, PubMed, and Web of Science. By focusing on relevant keywords in titles and abstracts from articles published between 2018 and 2024, we initially found 571 sources.

**Results:**

Thirty-seven articles that met the inclusion criteria were analyzed in this study. The attributes of evidence included four themes: focus and immersion, coding and understanding, arranging layers and algorithms, and implementing within the process of use cases to modify recommendations. Antecedents, unclear systems and communication, insufficient data management knowledge and support, and compound challenges can lead to suffering and risky caregiving tasks. Applying deep learning techniques enables nurses to simulate scenarios, predict outcomes, and plan care more precisely. Embracing deep learning equipment allows nurses to make better decisions. It empowers them with enhanced knowledge while ensuring adequate support and resources essential for caregiver and patient well-being. Access to necessary equipment is vital for high-quality home healthcare.

**Conclusion:**

This study provides a clearer understanding of the use of deep learning in nursing and its implications for nursing practice. Future research should focus on exploring the impact of deep learning on healthcare operations management through quantitative and qualitative studies. Additionally, developing a framework to guide the integration of deep learning into nursing practice is recommended to facilitate its adoption and implementation.

## Background

Nursing is crucial for supporting patients with chronic illness or those in need of ongoing care, whether they are healthy or awaiting continuous long-term assistance. By promoting health, preventing disease, treating illnesses, and facilitating rehabilitation, nurses contribute significantly to the well-being of individuals, families, and communities. This requires delivering services that prioritize suitability and enhance quality of life, drawing upon scientific principles and nursing expertise [1–3]. Technology integration is fundamental for advancing the caregiving process, as it empowers nurses to offer more efficient and streamlined care. By incorporating technology into caregiving and treatment, nurses can provide more effective assistance, ensuring that patients receive the highest standard of care. Despite technological advancements, compassionate care—often referred to as the human touch—remains paramount. Technology can further augment this compassionate care, ensuring that patient needs are met efficiently and emotionally [1, 4–6].

In recent years, there has been a growing emphasis on integrating AI technology into nursing practices, marking a significant milestone in adopting AI in patient care [2, 6–8]. This movement underscores the importance of deploying AI systems effectively within healthcare settings. Within this context, machine learning (ML) and DL are essential components. DL, a specialized branch of AI and ML, harnesses artificial neural networks (ANNs) with multiple layers, commonly known as “deep” networks. The pivotal role of DL within the broader AI landscape is evident, as it introduces advanced techniques that enhance the capabilities of intelligent systems while adhering to specific parameters and constraints. Inspired by the human brain, DL algorithms layer at understanding intricate patterns in extensive datasets, driving breakthroughs in areas such as computer vision, natural language processing, and speech recognition [1, 9–12]. As a subset of AI, ML and DL play a crucial role in advancing the development of intelligent systems and fostering innovation across various sectors. Examining AI functionalities and practical implementation strategies is vital for enhancing privacy measures and optimizing resource allocation in healthcare settings. However, while AI has promising outcomes, its implementation may pose unforeseen challenges and adverse effects, impacting medical treatment and healthcare delivery within hospitals [1, 12–15]. Furthermore, a pressing need exists to delve more deeply into the potential applications of AI in nursing and healthcare to enhance overall healthcare provision. It is essential to address pertinent issues such as bias and algorithmic considerations, which are integral to evaluating the efficacy and ethical implications of AI interventions. Ultimately, the prudent use of AI technology is essential for achieving outcomes aligned with the highest ethical standards and practices in nursing and healthcare [1, 14–17].

Numerous studies have explored integrating deep learning into healthcare systems, highlighting its potential to enhance nursing practices using advanced artificial intelligence tools. Moreover, deep learning algorithms empower nurses to analyze patient data for more effective decision-making, outcome prediction, and treatment planning. These methods expedite the identification of abnormalities in medical images and streamline documentation processes. Furthermore, deep learning contributes to safer medication management and facilitates remote patient monitoring, enabling nurses to intervene promptly. With its capacity for personalized care, deep learning holds promise for revolutionizing nursing practices, ultimately resulting in improved patient outcomes and healthcare delivery [16–21]. DL within artificial intelligence has garnered considerable attention in the contemporary era. However, navigating the operational complexities of ML and ANNs presents a distinct challenge, particularly within the nursing profession and healthcare providers. Establishing clear operational definitions and frameworks is essential for effectively integrating deep learning into nursing practice. Given the rapid evolution of AI, insights derived from current analyses may only be intermittently applicable to future practices, influenced by emerging features, requirements, and outcomes. Furthermore, in the realm of health science and technology, Morse’s approach, as applied by Zhida Shang, has been pivotal in understanding ‘artificial intelligence in nursing’ [22]. A significant limitation arises from the need for comprehensive studies that define, apply, trace origins, delineate characteristics, and outline the consequences of AI in nursing. Nonetheless, enhancing clarity and understanding of these concepts is crucial for advancing knowledge in nursing science. This research aims to explore the concept of deep learning within artificial intelligence as it pertains to nursing, utilizing Walker and Avant’s concept analysis method [23] to ensure a comprehensive and shared understanding. The study seeks to clarify the meaning and identify the defining attributes of artificial intelligence in the deep learning of artificial intelligence within nursing.

## Methods

### Concept analysis approach

In the field of nursing science, concept analysis is a crucial research tool that helps broaden understanding. It includes breaking down essential concepts into their basic parts, providing researchers with valuable insights. Through this approach, nursing experts refine definitions, make measurements precise and offer new viewpoints on things of interest. Concept analysis reveals the unique features of concepts that are vital in the ever-changing world of nursing science. It is essential to recognize that concepts in this field change over time. The procedure used in this concept analysis is based on Walker and Avant’s method [23], providing an evaluation framework for examining the concept’s definition, characteristics, antecedents, consequences, and scope, ensuring thorough understanding. Additionally, Morse’s analytical foundation approach facilitates the evaluation of ideas with clear boundaries, making it appropriate for this context [24]. Understanding DL aspect of AI in nursing requires clarifying terms often used interchangeably, which is crucial for advancing knowledge and developing theories. The application of DL in nursing converges multiple disciplines, resulting in a multi-dimensional concept. Current literature on AI in nursing needs to be more specific, with various interchangeable and poorly understood definitions, necessitating concept development, explanation, and comparison. The absence of a comprehensive definition for DL in AI within nursing is concerning, as future researchers might rely on standard dictionary definitions, leading to broader implications due to competing definitions. Thus, clarifying and differentiating these definitions using Walker and Avant’s method is essential. Therefore, concept analysis is an ongoing effort to maintain the evolving understanding of nursing science.

### Data collection

**Fig. 1:**
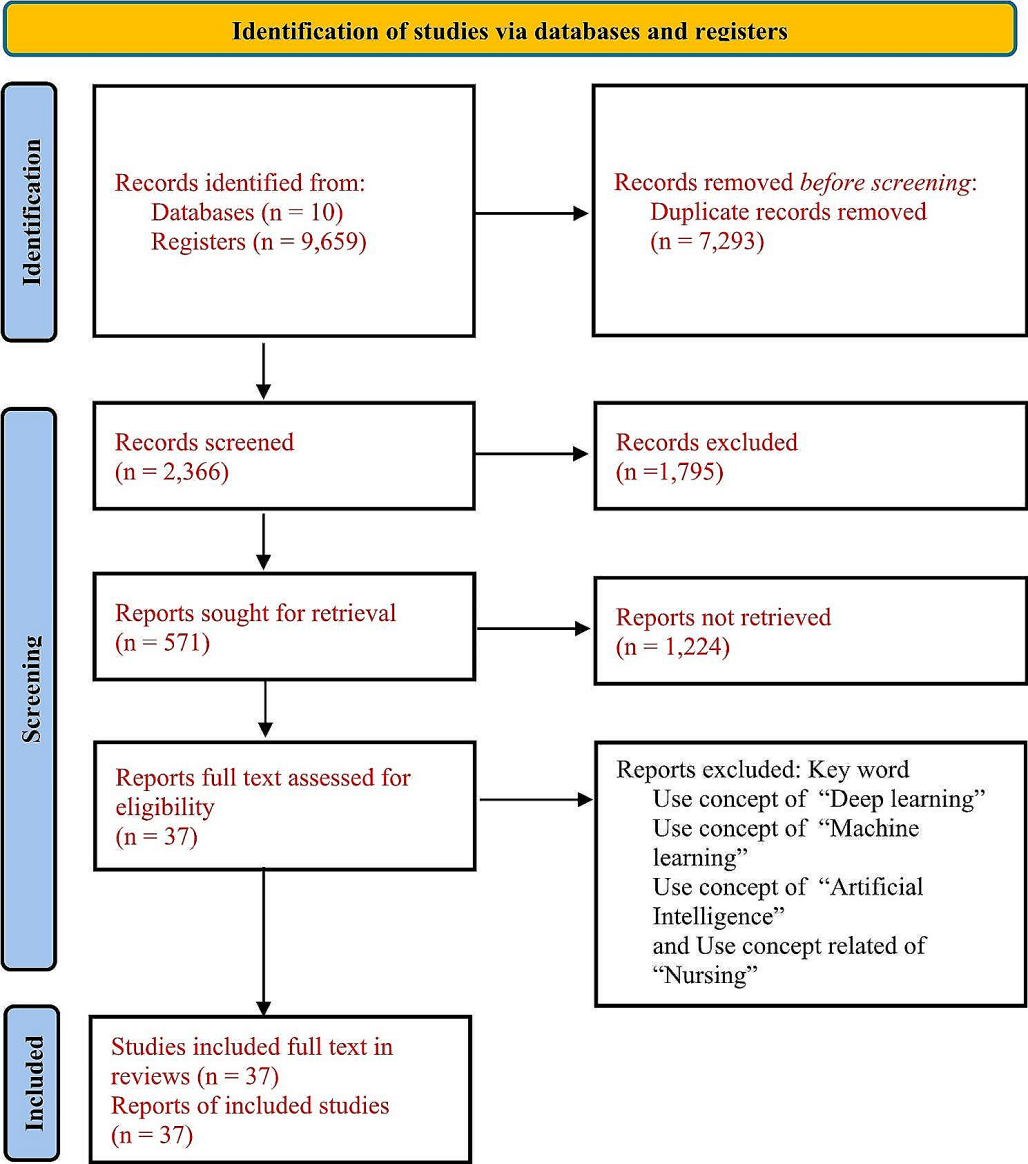
PRISMA diagram outlining the search strategy

This study used a systematic analytical approach. The study aimed to clarify the concept with keywords such as deep learning, machine learning, artificial intelligence, and nursing by determining its dimensions using the approach of Walker and Avant [23]. To thoroughly delve into the depth of the concept, an exhaustive search of the literature from 2018 to 2024 was meticulously conducted across a wide array of diverse databases employing rigorous Boolean techniques and MeSH terms [25, 26]. A total of 2,366 articles were screened, eventually yielding 571 relevant articles across ten databases: 181 from PubMed, 159 from ClinicalKey for Nursing, 135 from SpringerLink and Spinger Nature, 31 from Ovid, 24 from Scopus, 18 from ProQuest, 13 from CINAHL, five from Embase, three from BMC, and two from the Web of Science show in Table 1. Following the methodology advocated by Walker and Avant [23], the search strategy strategically employed Boolean techniques to collect pertinent information for concept analysis. Subsequently, the concept analysis method was meticulously executed, encompassing a series of steps: (1) selection of a concept, (2) determination of the aims or purposes of analysis, (3) determination of the defining attributes, and (4) identification of all uses of the concept. (5) Identify the antecedents and consequences. (6) Identify a model case, (7) identify borderline and related cases, and (8) define empirical referents. This systematic and rigorous approach ensures a comprehensive understanding of the concept’s dimensions and its implications within the field of nursing and its related domains. Finally, 36 articles were selected for analysis, and the PRISMA diagram is shown in Fig. 1 [27].

**Table 1:** The search strategies and results summary for each database

Database	Search Strategy	Filters	Eligible	Results
PubMed	((“Deep learning” AND (“Machine learning”) AND “Artificial Intelligence”) AND (“Nursing”) AND (“Concept Analysis”))	Publication date: 2018–2024, English language, Systematic reviews	181	2
ClinicalKey for Nursing	((“Deep learning” AND (“Machine learning”) AND “Artificial Intelligence”) AND (“Nursing”) AND (“Concept Analysis”))	Publication date: 2018–2024, English language, Systematic reviews	159	3
Springerlink and Spinger Nature,	((“Deep learning” AND (“Machine learning”) AND “Artificial Intelligence”) AND (“Nursing”) AND (“Concept Analysis”))	Publication date: 2018–2024, English language, Systematic reviews	135	4
Ovid	((“Deep learning” AND (“Machine learning”) AND “Artificial Intelligence”) AND (“Nursing”) AND (“Concept Analysis”))	Publication date: 2018–2024, English language, Systematic reviews	31	9
Scopus	((“Deep learning” AND (“Machine learning”) AND “Artificial Intelligence”) AND (“Nursing”) AND (“Concept Analysis”))	Publication date: 2018–2024, English language, Systematic reviews	24	4
ProQuest,	((“Deep learning” AND (“Machine learning”) AND “Artificial Intelligence”) AND (“Nursing”) AND (“Concept Analysis”))	Publication date: 2018–2024, English language, Systematic reviews	18	2
CINAHL	((“Deep learning” AND (“Machine learning”) AND “Artificial Intelligence”) AND (“Nursing”) AND (“Concept Analysis”))	Publication date: 2018–2024, English language, Systematic reviews	13	2
Embrace	((“Deep learning” AND (“Machine learning”) AND “Artificial Intelligence”) AND (“Nursing”) AND (“Concept Analysis”))	Publication date: 2018–2024, English language, Systematic reviews	5	5
BMC	((“Deep learning” AND (“Machine learning”) AND “Artificial Intelligence”) AND (“Nursing”) AND (“Concept Analysis”))	Publication date: 2018–2024, English language, Systematic reviews	3	3
Web of Science	((“Deep learning” AND (“Machine learning”) AND “Artificial Intelligence”) AND (“Nursing”) AND (“Concept Analysis”))	Publication date: 2018–2024, English language, Systematic reviews	2	3

## Results

### Selection of a concept

The review of the literature emphasizes the critical importance of integrating AI into healthcare, particularly within nursing. Collaborative efforts between AI and nursing teams can potentially enhance healthcare outcomes, as the promotion of AI education among nursing staff is vital for sustainable development. These studies offer valuable insights into the role of deep learning in healthcare, potentially strengthening our understanding of deep learning in AI applications. Therefore, the main objective of this research is to explore deep learning in artificial intelligence within the context of nursing science through concept analysis.

### Determine the aims or purposes of analysis

This study explored how deep learning in artificial intelligence can be applied in nursing, aiming to define its framework and relevance within the field. This highlights the significance of integrating these concepts into nursing practice to enhance patient care using modern technological advancements. Through an analysis of relevant activities and projects in nursing science, this research aims to elucidate the fundamental principles and defining attributes of deep learning in AI.

### Identifying all uses of the concept that you can discover

An onomasiological approach was used to define the concept across various contexts. Deep learning involves the theory and development of computer systems capable of tasks like speech recognition, learning, visual perception, mathematical computations, reasoning, problem-solving, decision-making, and language translation. Unlike traditional algorithms that focus narrowly, deep learning employs supervised or unsupervised machine learning techniques. These methods create and refine neural network models through multi-layered data representations and nonlinear transformations. Medical Subject Headings (MeSH) terms vary in specificity in nursing research and literature. There isn’t a single MeSH term combining “deep learning” and “artificial intelligence” in nursing. However, terms like “Artificial Intelligence,” “Machine Learning,” and “Neural Networks” collectively cover these concepts. These terms help researchers access relevant studies in nursing databases, supporting advancements in patient care, clinical decision-making, and nursing education [25, 26].

Artificial intelligence was first coined by John McCarthy in 1956 at Dartmouth College. Since then, AI has evolved significantly, emphasizing technological foundations and cognitive resonance with human intelligence. This evolution led to machine learning paradigms introducing models like Artificial Neural Networks (ANNs) [1, 10–12]. Deep learning, initially conceptualized by Dechter in 1986, constructs complex functions through layered neural networks. These architectures mimic hierarchical processing in biological brains with input, hidden, and output layers. Techniques like backpropagation refine mathematical operations to discern patterns and insights from extensive datasets [28]. This narrative begins with AI’s inception and traces its evolution to deep learning [1, 10–12]. A detailed onomasiological analysis shows that “deep learning” involves immersion, focus, and profound understanding, contrasting with superficial notions. This cognitive engagement parallels human brain functions, supporting memory and learning pathways. In education and computer science, deep learning promotes critical thinking, problem-solving, and practical knowledge application [29, 30]. Projects and teamwork encourage deep subject exploration and creative solutions. Computationally, deep learning enhances performance through dataset analysis [31, 32]. This mirrors hierarchical brain learning with supervised, unsupervised, or reinforcement methods to absorb knowledge [9–12, 33–35].

### Determine the defining attributes

Deep learning holds promise for transforming nursing practice by enhancing clinical decision-making, improving patient monitoring, and enabling predictive analytics. By leveraging these advanced technologies, nurses can provide more accurate, timely, and personalized care, ultimately improving patient outcomes and operational efficiency in healthcare settings. As the field evolves, ongoing research and collaboration between data scientists and healthcare professionals will be crucial to fully realizing the benefits of deep learning in nursing. In-depth learning refers to in-depth work that involves an input process and has functions in both analysis and algorithmic processing. It is characterized by its capacity to learn hierarchical data representations, progressing from fundamental concepts to more abstract ones. This hierarchical learning enables deep models to apprehend intricate patterns by capturing features at varying levels of abstraction. Training deep learning models necessitates extensive labeled datasets, commonly called big data, facilitating robust generalization to new examples and enhancing overall performance [9–12]. The versatility of deep learning is evident in its wide-ranging applications across domains such as image and speech recognition and natural language processing, demonstrating its ability to perform diverse tasks. Specialized architectures such as ANNs can optimize performance by tailoring the model structures to handle specific data types and tasks effectively. Additionally, deep learning fosters autonomous knowledge acquisition, enabling machines to learn and apply knowledge from data without explicit programming autonomously. Compared with their shallow counterparts, deep networks often exhibit efficiency in approximating functions, achieving comparable results with fewer computational resources. As a subset of machine learning and artificial intelligence, deep learning aims to emulate human brain networks, enabling machines to learn and make decisions akin to human cognition. From a nursing science perspective, numerous studies have demonstrated the potential application of deep learning in research [9–12, 30].

When delving into the intricacies of deep learning, specific components emerge. This entails synthesizing various works and organizing them into a structured framework harmonized with a formal control system to enhance nursing care. Focus, immerse, code, understand, arrange layers and algorithms, and implement within the use process. This involves (1) **focusing and immersing** individuals in the central point of interest or attention and thoroughly engaging individuals in an activity and implementing a tactile tool or a system that requires manual manipulation to initiate the process within a use case scenario—a series of actions to achieve a specific outcome. Focusing or visualizing the original knowledge, which in this context is deep learning, is crucial. Participants must be sincerely interested and immersed in the activity, aligning with predefined objectives or goals to foster commitment and engagement. This approach forms the cornerstone for success in any endeavor. (2) **Coding and understanding** are foundational pillars in the intricate landscape of deep learning, particularly in enhancing nursing care. In deep learning, coding involves meticulously arranging words, letters, or symbols to encapsulate messages, whether for encryption or concise communication. This process, which may utilize numerical, alphabetical, or symbolic systems, encodes information conducive to efficient comprehension and analysis. On the other hand, understanding delves into the profound assimilation and interpretation of conveyed meaning, facilitating informed decision-making. This comprehensive grasp of information is essential for nurses as they navigate the complexities of patient care, encompassing direct clinical interventions and the broader implications of illness and healthcare practices. Through deep understanding, nurses can adeptly decipher the intricacies of patient conditions, treatment plans, and the ever-evolving healthcare landscape, ultimately fostering optimal patient outcomes and enhancing healthcare delivery. (3) **Arranging layers and algorithms** involves structuring data akin to distinct material or thin sheet levels and utilizing mathematical instructions or rules to guide problem-solving. This process encompasses organizing layers based on specific instructions or mathematical principles, allowing computers to comprehend and process information effectively. This attribute also entails utilizing tools to execute tasks based on predefined instructions and algorithms. For instance, learning algorithms adeptly capture constraints involving a limited number of variables while arranging layers, and following prescribed algorithms ensures systematic processing of information to address the given problem. Finally, implementation involves employing tools following predefined instructions to achieve results aligned with the established algorithm. (4) In **the implementation process within the use case**, meticulous attention is directed toward practice, employing a systematic approach reminiscent of principles to yield favorable outcomes. This approach emphasizes the importance of evidence-based practices, drawing upon empirical evidence to enhance the quality of care and ultimately improve patient outcomes. Implementation strategies may involve the utilization of simulations or carefully crafted designs aimed at gaining a comprehensive understanding of patient needs and intricacies, facilitating the effective planning and delivery of care. By adhering to evidence-based practices and leveraging innovative tools such as simulations, healthcare professionals can navigate the complexities of patient care with precision and compassion, ensuring optimal outcomes and patient satisfaction. However, with the advancement of AI technology, the intricacies of utilizing deep learning in nursing have become increasingly significant. Investigating the role of AI is crucial, particularly in nurse registration. This approach involves developing a process to monitor advancements, encompassing health trends and nursing research [9–12, 21, 31].

### Identify a model case

The idea of understanding deep learning can be divided into four parts: focusing, understanding code, organizing layers and algorithms, and applying it in real-life situations. Now, picture a community health nurse who plans a home visit (home health care [32, 36]) of a 98-year-old grandfather unable to leave his bed. The nurse assisted with daily tasks using a scale called the Barthel ADL Index, where a score of 1 out of 20 is typical. Additionally, family members, such as grandchildren-in-law, are waiting to assist, perhaps with tasks such as changing diapers twice a day. Imagine a community health nurse preparing for a home visit to care for a 98-year-old grandfather who is bedridden. Nurses rely on tools such as the Barthel ADL Index to assess the grandfather’s needs, which are typically just 1 out of 20. Family members, such as grandchildren-in-law, are also involved in caregiving, aiding with tasks such as changing diapers and scheduling visits. However, nurses face the challenge of understanding grandfathers’ complex health issues to provide tailored assistance effectively. To address this, the nurse delves into the patient’s records, utilizing electronic health records (EHR) systems to access crucial information for diagnosis and treatment. Mastery of medical applications and computer programs is also essential for medication management and accurate interpretation of test results. Additionally, nurses embrace telehealth tools, such as video calls, to facilitate efficient communication with healthcare teams, overcoming geographical barriers. However, ensuring patient privacy and data security remains paramount when utilizing these technologies. Organizing care plans and applying algorithms to meet the grandfather’s specific needs are crucial in caregiving. Understanding these principles allows nurses to provide systematic and efficient care while safeguarding patient information from unauthorized access or tampering. In essence, by integrating deep learning concepts into caregiving practices, nurses can increase the standard of home visits for older patients, such as grandfathers. This holistic approach ensures adherence to guidelines and maximizes efficiency and convenience, leveraging the support of family members for an optimal caregiving experience [17]. The deep learning prediction model and the resulting risk stratification score may prove helpful in clinical decision-making under time-sensitive and resource-constrained environments [37–44].

### Identify contrary cases and borderline cases

The visiting nurse received an urgent call from a concerned patient, a 67-year-old individual seeking assistance from their 85-year-old grandmother who had recently suffered a debilitating stroke three months prior. The grandmother was bedridden, her movements were restricted, and her ability to sense her surroundings greatly diminished. Complicating matters, the feeding tube she relied upon often became dislodged, requiring frequent readjustments and careful monitoring. Upon arrival, the nurse encountered a distressing scene. Essential equipment needed to be included or completed, forcing the nurse to improvise with makeshift solutions. This hindered the efficiency of care and posed risks to the grandmother’s well-being. Adding to the situation’s complexity was the psychological and economic stress experienced by the family caregivers. Individuals constantly tending to meet the needs of elderly loved ones with serious health issues exacted a heavy toll, both emotionally and financially. The demands of continuous care left caregivers exhausted and overwhelmed, exacerbating the challenges faced by nurses upon arrival. Furthermore, the need for premade equipment added another layer of complications. Procuring these items often involved lengthy delays, further impeding the provision of timely and effective care—the strain of navigating these logistical hurdles not only exacerbated the already tense atmosphere. During these challenges, it became evident that the standard of care fell short of what was necessary for the grandmother’s well-being. Despite the caregivers’ best efforts, the lack of adequate preparation and support left the grandmother vulnerable and in need of more comprehensive care. This scenario underscores the critical importance of recognizing and addressing the psychological and economic stress experienced by family caregivers. Providing patients with adequate support and resources is essential for ensuring the well-being of both the caregiver and the care recipient. Additionally, ensuring that healthcare professionals access the necessary equipment and resources is vital for delivering high-quality care in home healthcare settings.

### Identify the antecedents and consequences

When exploring the antecedents of DL in nursing, it becomes evident that nurses across various care settings face overwhelming challenges and demands that directly impact patient care. These antecedents include the repetitive nature of caregiving tasks, inadequate knowledge of data management, and insufficient support. Nurses in both care facilities and community settings juggle multiple roles amidst ambiguity in systems and communication, leading to risky and repetitive caregiving tasks that contribute to heightened stress and fatigue. The lack of knowledge in data management significantly hinders the effective utilization of the nursing process, while insufficient support and resources exacerbate these challenges, impeding effective communication and continuous learning. This intensifies feelings of isolation and frustration among caregivers, underscoring the complexities inherent in caregiving and highlighting the need for enhanced support and training in data management and communication [17, 18]. Consequently, integrating DL in nursing can lead to several beneficial outcomes. Enhanced support and training foster trust and precision in evaluating and planning nursing care, resulting in a more explicit, outcome-oriented process. Nurses can seamlessly apply DL technology within their practice, intertwining it with various related arts such as simulating complex scenarios, crafting intricate decision trees, and predicting outcomes through advanced algorithms like deep belief networks and multilayer perceptron’s. This integration addresses existing challenges and advances the profession by enhancing the accuracy and efficiency of patient care, ultimately leading to better patient outcomes and a more efficient healthcare system [17, 18].

### Define the empirical referents

Directly connected to the theory behind DL, we see that whatever can be measured is retained. This is evident when we try receiving and interpreting signals. In nursing, careful attention is given to science. We collected various data for analysis, aiming for meaningful results. Understanding these principles empowers nurses to provide well-organized, impactful data-based care. It has been noted that what can be measured there is kept. This is noticeable in the trial of the reception of the receipt signal. Care in the scientific direction of nursing may be all forms of data recorded to be analyzed and interpreted appropriately, and possible results may be obtained. Understanding these principles helps nurses provide systematic, effective care based on data.

## Discussion

This approach offers a systematic approach to refining one’s understanding of complex concepts, such as DL, by exploring various qualities and elements. DL, a subset of ML and AI, employs artificial neural networks to process vast datasets, revolutionizing industries such as healthcare and finance. Its multilayered architecture enables precise simulation and outcome prediction and promising enrichment of nursing and patient care across various domains. Utilizing Walker and Avant’s conceptual framework [23], critics of the DL concept typically follow eight steps; a contemporary concept widely applied today entails a thorough analysis to enhance functionality. Understanding its application in healthcare is crucial for nurses, given DL’s proficiency in learning hierarchical data representations through extensive labeled datasets, thus achieving robust performance across various tasks. DL leverages specialized architectures such as ANNs and transformers to optimize performance for specific tasks, promote automated knowledge acquisition and facilitate learning from explicit programming of data sans. Deep networks efficiently estimate functions, delivering comparable results with fewer computational resources [17, 18]. DL analysis entails meticulous data collection and investigation to delve into layer-by-layer data representations, necessitating large datasets. DL models showcase versatility in recognizing images, controlling specialized natural language features, and promoting autonomous knowledge acquisition. Moreover, DL fosters research efficiency by illuminating fundamental principles and structures in various applications, particularly in healthcare settings, with profound impact. The aim of deep learning concept analysis is to refine its functional scope and clarify and differentiate concepts to pave the way for future understanding and measurement. Systematic reviews help delineate the definition and scope of deep learning, enhancing the precision and comprehensiveness of its application [9–12].

This literature review explores DL within the AI, highlighting its significance and unique characteristics. DL stands at the forefront of transformative technologies, continually enhancing machines’ data processing and comprehension capabilities. By leveraging advancements in computational resources and methodologies, DL has the potential to revolutionize various industries. As a subset of AI and machine learning, DL replicates human-like abilities in pattern recognition and decision-making, utilizing artificial neural networks inspired by the human brain’s structure. Its intricate, multilayered architecture facilitates sophisticated data analysis, offering advantages over traditional machine-learning techniques [17, 18]. Despite these efficiency gains, DL methods require substantial computational power and time due to their complex neural networks and the handling of large datasets. The discussion section should prioritize core concepts such as integrating DL into nursing care and its technical foundations rather than solely focusing on its technological and mechanical applications. While the current overview provides a broad perspective on DL and its implications for nursing practice, a more profound exploration of specific challenges, opportunities, and future directions would enhance its depth and relevance. This deeper analysis can elucidate how DL can effectively enhance patient care and nursing outcomes while addressing the ethical and practical considerations accompanying its implementation in healthcare settings. Furthermore, a detailed examination of DL’s implications in nursing could illuminate innovative methodologies and approaches that leverage its capabilities to improve diagnostic accuracy, treatment planning, and patient monitoring. Such an expanded discussion would underscore DL’s transformative potential in healthcare and contribute a clearer understanding of the strategic initiatives necessary for responsible and effective integration into nursing practice. These insights can inform policies and practices that support the ethical deployment of DL, ensuring alignment with patient-centered care and advancing nursing excellence in the future. Integrating AI in nursing requires enhancing nurses’ qualifications and education to comprehend AI’s capabilities and limitations, complemented by informational campaigns to equip nurses with essential skills. DL technology is intricately layered in nursing applications to encompass understanding healthcare contexts, collecting and preprocessing diverse data, developing specialized algorithms, and integrating models into clinical workflows. Implementation strategies involve collaborative efforts to align AI with nursing standards, integrate it into existing infrastructure, provide adequate training, and ensure ethical usage. These initiatives promise to enhance patient care, streamline workflows, and address ethical, legal, and societal concerns, significantly advancing nursing practice [37–45].

### Limitations

The study’s limitation is its dependence on theoretical analysis without empirical validation, which diminishes its applicability to real-world nursing education and highlights a significant research gap. Empirical validation could have improved the understanding of deep learning in nursing, offered practical insights into its educational implications, identified implementation challenges, and confirmed theoretical constructs. The introduction highlights the limited number of studies in AI and notes that the field is continually evolving with new deep learning approaches. This scarcity and the rapidly changing nature of AI make in-depth exploration difficult, emphasizing the need for empirical studies to bridge this gap and ensure theoretical models are relevant in practical contexts.

## Conclusion

This study enhances the understanding of the application of deep learning in nursing care and underscores its potential implications for nursing practice. Moving forward, future research needs to further explore the impact of deep learning on healthcare operations management through quantitative and qualitative studies. Researchers can provide valuable insights into the transformative potential of deep learning technology by examining how deep learning technologies can optimize various aspects of healthcare delivery, such as resource allocation and patient monitoring. Moreover, developing a comprehensive framework is crucial for effectively integrating deep learning into nursing practice. This framework should not only address technical considerations but also encompass ethical, legal, and regulatory aspects to ensure the responsibility and ethical use of AI in healthcare settings. Additionally, future research should acknowledge and address the limitations of this study, such as potential biases in article selection or analysis methodologies, to foster a more robust and nuanced understanding of the role of deep learning in nursing care. Nurses can augment their decision-making capabilities by embracing deep learning technologies, enhancing patient care outcomes, and streamlining clinical workflows. As we continue to explore and harness the potential of AI in healthcare, collaboration between researchers, clinicians, policymakers, and industry stakeholders will be essential to drive innovation and facilitate the widespread adoption of deep learning in nursing practice.

### Recommendations

The exploration of deep learning as a subset of artificial intelligence and machine learning underscores its potential to revolutionize nursing practices. While the literature review reveals the limited presence of deep learning applications in nursing research, particularly in medical domains and interdisciplinary fields, the identified gap highlights an opportunity for integration. Inspired by the human brain’s neural networks, deep learning offers advantages in recognizing patterns and making decisions, showcasing its versatility in handling diverse datasets. The study’s findings emphasize the need for nurses to embrace deep learning, empowering them with enhanced knowledge and decision-making capabilities. By integrating deep learning into nursing practices, nurses could be equipped with tools to make more informed decisions, especially in diagnostic nursing, thereby improving healthcare operation assessments and reducing the risk of medical disputes. Moving forward, future research proposals and the development of a framework are recommended to explore the impact of deep learning on healthcare operations management. Such initiatives aim to promote safety and enhance the overall quality of life for patients and communities globally, reflecting the transformative potential of deep learning as technology continues to advance. Preparing nurses for AI deep learning in healthcare integration requires updating their qualifications, educating them about AI capabilities and limitations, and conducting nursing care-specific research. The involvement of researchers, policymakers, and guideline developers ensures alignment with standards, while discussions on ethical, legal, and societal implications are crucial for stakeholder participation and advancement.

## Data Availability

DeclarationsUnder ‘Declarations,’ this study involved a retrospective review of records and did not include any direct interaction with patients by the research team. The data and materials used or produced throughout this study have been included in this manuscript”.

## Declarations

Under ‘Declarations,’ this study involved a retrospective review of records and did not include any direct interaction with patients by the research team. The data and materials used or produced throughout this study have been included in this manuscript”.

## Abbreviations

AI: Artificial Intelligence
ANNs: Artificial Neural Networks
CDS: Clinical Decision Support
CINAHL: Cumulative Index to Nursing & Allied Health Literature
DL: Deep Learning
ML: Machine Learning
MeSH: Medical Subject Headings
PRISMA: Preferred Reporting Items for Systematic Reviews and Meta Analyses
